# 1-(3,4-Dihydroxy­phen­yl)-2-(4-hydroxy­phen­yl)ethanone

**DOI:** 10.1107/S1600536809049393

**Published:** 2009-12-04

**Authors:** Zhu-Ping Xiao, Shou-Fu Yi, Gao-Yu Ou, Fang Zhang, Jian Zhu

**Affiliations:** a Key Laboratory of Plant Resources Conservation and Utilization (Jishou University), College of Hunan Province, Jishou University, Jishou 416000, People’s Republic of China; b College of Chemistry & Chemical Engineering, Jishou University, Jishou 416000, People’s Republic of China

## Abstract

The title compound, C_14_H_12_O_4_, is a deoxy­benzoin derivative in which the dihedral between the carbonyl group and the catechol unit is 5.99 (3)°. The dihedral angle between the two benzene rings is 60.26 (13)°. In the crystal structure, inter­molecular O—H⋯O hydrogen bonds connect mol­ecules, forming a two-dimensional network. In addition, weak inter­molecular C—H⋯O hydrogen bonds and C—H⋯π contacts further stabilize the crystal structure.

## Related literature

For synthetic applications of deoxy­benzoin compounds, see: Xiao *et al.* (2007*a* ▶, 2008*a* ▶). For natural occurences of these compounds. see: Kiuchi *et al.* (1990 ▶); Niwa *et al.* (1999 ▶); Sanduja *et al.* (1985 ▶). For their biological activity, see: Papoutsi *et al.* (2007 ▶); Xiao *et al.* (2007*b* ▶, 2008*b* ▶); Parmar *et al.* (1996 ▶). For a related structure, see: Xiao & Xiao (2008*c* ▶).
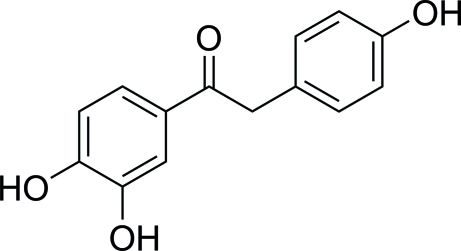


## Experimental

### 

#### Crystal data

C_14_H_12_O_4_
*M* *_r_* = 244.24
Triclinic, 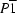

*a* = 5.7073 (11) Å
*b* = 9.3464 (19) Å
*c* = 11.202 (2) Å
α = 100.112 (9)°
β = 94.792 (9)°
γ = 100.625 (9)°
*V* = 573.99 (19) Å^3^
*Z* = 2
Mo *K*α radiation
μ = 0.10 mm^−1^
*T* = 296 K
0.30 × 0.20 × 0.20 mm

#### Data collection

Bruker SMART APEX CCD diffractometer
Absorption correction: multi-scan (*SADABS*; Sheldrick, 1996 ▶) *T*_min_ = 0.970, *T*_max_ = 0.980
3179 measured reflections
2214 independent reflections
1756 reflections with *I* > 2σ(*I*)
*R*_int_ = 0.028

#### Refinement

*R*[*F* ^2^ > 2σ(*F* ^2^)] = 0.055
*wR*(*F* ^2^) = 0.172
*S* = 1.06
2214 reflections
166 parameters
H-atom parameters constrained
Δρ_max_ = 0.26 e Å^−3^
Δρ_min_ = −0.32 e Å^−3^

### 

Data collection: *SMART* (Bruker, 2007 ▶); cell refinement: *SAINT* (Bruker, 2007 ▶); data reduction: *SAINT*; program(s) used to solve structure: *SHELXS97* (Sheldrick, 2008 ▶); program(s) used to refine structure: *SHELXL97* (Sheldrick, 2008 ▶); molecular graphics: *SHELXTL* (Sheldrick, 2008 ▶); software used to prepare material for publication: *SHELXL97*.

## Supplementary Material

Crystal structure: contains datablocks global, I. DOI: 10.1107/S1600536809049393/lh2957sup1.cif

Structure factors: contains datablocks I. DOI: 10.1107/S1600536809049393/lh2957Isup2.hkl

Additional supplementary materials:  crystallographic information; 3D view; checkCIF report

## Figures and Tables

**Table 1:** Hydrogen-bond geometry (Å, °)

*D*—H⋯*A*	*D*—H	H⋯*A*	*D*⋯*A*	*D*—H⋯*A*
O1—H1⋯O4^i^	0.82	1.94	2.744 (2)	168
O2—H2⋯O3^ii^	0.82	1.91	2.7274 (19)	171
O4—H4⋯O2^iii^	0.82	2.00	2.772 (2)	158
C3—H3⋯O3^ii^	0.93	2.53	3.191 (2)	129
C11—H11⋯*Cg*1^iv^	0.93	2.85	3.635 (2)	143

Symmetry codes: (i) 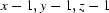; (ii) 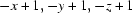; (iii) 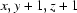; (iv) 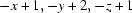. *Cg*1 is the centroid of the C1–C6 ring.

**Fig. 1.**
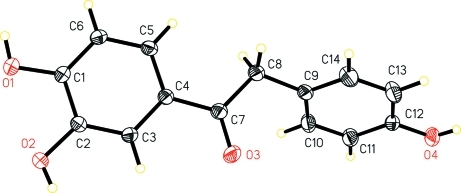
Molecular structure of the title compound, showing the atom-numbering scheme. Displacement ellipsoids are drawn at the 30% probability level.

**Fig. 2.**
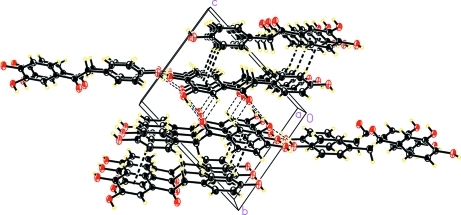
Part of the crystal structure of (I) with hydrogen bonds indicated by thin dashed lines and C—H···π contacts shown as thick dashed lines.

## supplementary crystallographic information

### Comment

Deoxybenzoin compounds are intermediates in the syntheses of isoflavones (Xiao *et al.*, 2008*a*; Xiao *et al.*, 2007*a*) and are found in several plants, such as Glycyrrhiza *sp*., Trifolium subterraneum and Ononis spinosa, and marine sources (Kiuchi *et al.*, 1990; Niwa *et al.*, 1999; Sanduja *et al.*, 1985). Many deoxybenzoin compounds have shown significant estrogen receptor modulatory, urease inhibitory, antimicrobial and antiviral properties (Papoutsi *et al.*, 2007; Xiao *et al.*, 2007*b*; Xiao *et al.*, 2008*b*; Parmar *et al.*, 1996). As part of our work involving the synthesis of a series of deoxybenzoin derivatives for urease inhibitory activity screening we report herein the crystal structure of the title deoxybenzoin derivative (I).

The molecular structure of the title compound is shown in Fig. 1. The two benzene rings form a dihedral angle of 60.26 (13) °. The carbonyl group forms a dihedral angle of 5.99 (3) ° with the catechol moiety [O1/O2/C1-C6] while a similar angle is  1.95 (13)°  in our previous related crystal structure (Xiao *et al.*, 2008*c*). In the crystal structure, intermolecular O—H···O  connect molecules to form a two-dimensional network. In addition, weak intermolecular C—H···O hydrogen bonds and C—H···π contacts further stabilize the crystal structure (see Fig. 2).

### Experimental

0.55 g (5 mmol) of catechol and 0.76 g (5 mmol) of *p*-hydroxyphenylacetic acid were dissolved into 10 ml of fresh distilled BF_3_.Et_2_O. The mixture was stirred and heated on an oil bath at 353 K for about 3 h. After cooling, the contents were poured into 150 ml of ice-cold aqueous sodium acetate (w % = 10%) with stirring. Then, the precipitate was filtered and washed three times with water. The resulting solid was crystallized from methanol-water to give colorless blocks of (I) suitable for single-crystal structure determination.

### Refinement

All H atoms were placed in geometrically idealized positions and constrained to ride on their parent atoms with C—H of 0.93 Å for the aromatic atoms, 0.97 Å for the CH_2_ groups and 0.82 Å for the OH groups. *U*_iso_(H) values were set at 1.2 times *U*_eq_(C) for aromatic C and CH_2_ groups and 1.5 times *U*_eq_(O) for O—H groups.

### Crystal data

**Table:**

C_14_H_12_O_4_	*Z* = 2
*M**_r_* = 244.24	*F*(000) = 256
Triclinic, *P*1	*D*_x_ = 1.413 Mg m^−^^3^
Hall symbol: -P 1	Mo *K*α radiation, λ = 0.71073 Å
*a* = 5.7073 (11) Å	Cell parameters from 1785 reflections
*b* = 9.3464 (19) Å	θ = 2.0–26.0°
*c* = 11.202 (2) Å	µ = 0.10 mm^−^^1^
α = 100.112 (9)°	*T* = 296 K
β = 94.792 (9)°	Block, colorless
γ = 100.625 (9)°	0.30 × 0.20 × 0.20 mm
*V* = 573.99 (19) Å^3^	

### Data collection

**Table:**

Bruker SMART APEX CCD diffractometer	2214 independent reflections
Radiation source: fine-focus sealed tube	1756 reflections with *I* > 2σ(*I*)
graphite	*R*_int_ = 0.028
φ and ω scans	θ_max_ = 26.0°, θ_min_ = 1.9°
Absorption correction: multi-scan (*SADABS*; Sheldrick, 1996)	*h* = −7→6
*T*_min_ = 0.970, *T*_max_ = 0.980	*k* = −11→6
3179 measured reflections	*l* = −13→13

### Refinement

**Table:**

Refinement on *F*^2^	Primary atom site location: structure-invariant direct methods
Least-squares matrix: full	Secondary atom site location: difference Fourier map
*R*[*F*^2^ > 2σ(*F*^2^)] = 0.055	Hydrogen site location: inferred from neighbouring sites
*wR*(*F*^2^) = 0.172	H-atom parameters constrained
*S* = 1.06	*w* = 1/[σ^2^(*F*_o_^2^) + (0.1062*P*)^2^ + 0.1004*P*] where *P* = (*F*_o_^2^ + 2*F*_c_^2^)/3
2214 reflections	(Δ/σ)_max_ < 0.001
166 parameters	Δρ_max_ = 0.26 e Å^−^^3^
0 restraints	Δρ_min_ = −0.31 e Å^−^^3^

### Special details

**Table:**

Geometry. All e.s.d.'s (except the e.s.d. in the dihedral angle between two l.s. planes) are estimated using the full covariance matrix. The cell e.s.d.'s are taken into account individually in the estimation of e.s.d.'s in distances, angles and torsion angles; correlations between e.s.d.'s in cell parameters are only used when they are defined by crystal symmetry. An approximate (isotropic) treatment of cell e.s.d.'s is used for estimating e.s.d.'s involving l.s. planes.
Refinement. Refinement of *F*^2^ against ALL reflections. The weighted *R*-factor *wR* and goodness of fit *S* are based on *F*^2^, conventional *R*-factors *R* are based on *F*, with *F* set to zero for negative *F*^2^. The threshold expression of *F*^2^ > σ(*F*^2^) is used only for calculating *R*-factors(gt) *etc*. and is not relevant to the choice of reflections for refinement. *R*-factors based on *F*^2^ are statistically about twice as large as those based on *F*, and *R*- factors based on ALL data will be even larger.

### Fractional atomic coordinates and isotropic or equivalent isotropic displacement parameters (Å^2^)

**Table:**

	*x*	*y*	*z*	*U*_iso_*/*U*_eq_	
C1	0.0231 (3)	0.4370 (2)	0.17089 (17)	0.0357 (4)	
C2	0.2221 (3)	0.4285 (2)	0.24884 (17)	0.0342 (4)	
C3	0.2784 (3)	0.5201 (2)	0.36129 (17)	0.0357 (5)	
H3	0.4123	0.5140	0.4120	0.043*	
C4	0.1374 (3)	0.62287 (19)	0.40118 (17)	0.0335 (4)	
C5	−0.0606 (3)	0.6309 (2)	0.32327 (18)	0.0364 (5)	
H5	−0.1561	0.6986	0.3480	0.044*	
C6	−0.1164 (3)	0.5392 (2)	0.20949 (18)	0.0382 (5)	
H6	−0.2490	0.5460	0.1582	0.046*	
C7	0.2107 (4)	0.7192 (2)	0.52317 (18)	0.0377 (5)	
C8	0.0779 (4)	0.8423 (2)	0.56251 (19)	0.0434 (5)	
H8A	0.0603	0.8942	0.4956	0.052*	
H8B	−0.0821	0.7977	0.5769	0.052*	
C9	0.1937 (3)	0.9545 (2)	0.67514 (18)	0.0376 (5)	
C10	0.4194 (4)	1.0415 (2)	0.67929 (19)	0.0464 (5)	
H10	0.5049	1.0265	0.6126	0.056*	
C11	0.5210 (4)	1.1498 (2)	0.77965 (19)	0.0466 (5)	
H11	0.6730	1.2070	0.7802	0.056*	
C12	0.3970 (3)	1.1730 (2)	0.87899 (17)	0.0375 (5)	
C13	0.1733 (4)	1.0874 (2)	0.8778 (2)	0.0497 (6)	
H13	0.0887	1.1019	0.9449	0.060*	
C14	0.0751 (4)	0.9795 (2)	0.7761 (2)	0.0497 (6)	
H14	−0.0763	0.9220	0.7760	0.060*	
O1	−0.0188 (3)	0.34464 (17)	0.06112 (13)	0.0505 (4)	
H1	−0.1572	0.3388	0.0313	0.076*	
O2	0.3534 (2)	0.32578 (15)	0.20545 (12)	0.0420 (4)	
H2	0.4435	0.3133	0.2620	0.063*	
O3	0.3760 (3)	0.70119 (18)	0.59031 (14)	0.0609 (5)	
O4	0.5023 (3)	1.28604 (17)	0.97474 (14)	0.0507 (4)	
H4	0.4249	1.2818	1.0329	0.076*	

### Atomic displacement parameters (Å^2^)

**Table:**

	*U*^11^	*U*^22^	*U*^33^	*U*^12^	*U*^13^	*U*^23^
C1	0.0337 (10)	0.0355 (9)	0.0340 (10)	0.0061 (7)	−0.0017 (7)	0.0002 (8)
C2	0.0314 (10)	0.0336 (9)	0.0374 (10)	0.0096 (7)	0.0032 (7)	0.0034 (8)
C3	0.0340 (10)	0.0344 (9)	0.0373 (10)	0.0113 (8)	−0.0047 (8)	0.0028 (8)
C4	0.0343 (10)	0.0291 (9)	0.0366 (10)	0.0096 (7)	−0.0001 (8)	0.0036 (7)
C5	0.0319 (10)	0.0337 (9)	0.0433 (11)	0.0123 (7)	0.0005 (8)	0.0028 (8)
C6	0.0317 (10)	0.0392 (10)	0.0412 (11)	0.0101 (8)	−0.0056 (8)	0.0027 (8)
C7	0.0404 (11)	0.0330 (9)	0.0402 (11)	0.0141 (8)	−0.0016 (8)	0.0045 (8)
C8	0.0404 (11)	0.0383 (10)	0.0479 (12)	0.0162 (8)	−0.0047 (9)	−0.0052 (9)
C9	0.0377 (11)	0.0328 (9)	0.0413 (11)	0.0149 (8)	−0.0002 (8)	−0.0011 (8)
C10	0.0429 (12)	0.0534 (12)	0.0379 (11)	0.0079 (9)	0.0099 (9)	−0.0047 (9)
C11	0.0358 (11)	0.0521 (12)	0.0443 (12)	0.0021 (9)	0.0057 (9)	−0.0040 (9)
C12	0.0379 (11)	0.0371 (10)	0.0345 (10)	0.0116 (8)	−0.0008 (8)	−0.0030 (8)
C13	0.0486 (13)	0.0489 (12)	0.0470 (13)	0.0061 (10)	0.0174 (10)	−0.0046 (10)
C14	0.0391 (12)	0.0420 (11)	0.0610 (14)	0.0010 (9)	0.0114 (10)	−0.0039 (10)
O1	0.0472 (9)	0.0560 (9)	0.0417 (9)	0.0205 (7)	−0.0100 (7)	−0.0116 (7)
O2	0.0402 (8)	0.0456 (8)	0.0390 (8)	0.0206 (6)	−0.0024 (6)	−0.0043 (6)
O3	0.0755 (12)	0.0577 (10)	0.0467 (9)	0.0401 (8)	−0.0243 (8)	−0.0110 (7)
O4	0.0450 (9)	0.0559 (9)	0.0415 (8)	0.0076 (7)	0.0011 (6)	−0.0119 (7)

### Geometric parameters (Å, °)

**Table:**

C1—O1	1.348 (2)	C8—H8B	0.9700
C1—C6	1.388 (3)	C9—C14	1.373 (3)
C1—C2	1.396 (3)	C9—C10	1.383 (3)
C2—C3	1.369 (3)	C10—C11	1.378 (3)
C2—O2	1.372 (2)	C10—H10	0.9300
C3—C4	1.402 (3)	C11—C12	1.375 (3)
C3—H3	0.9300	C11—H11	0.9300
C4—C5	1.390 (2)	C12—C13	1.374 (3)
C4—C7	1.479 (3)	C12—O4	1.374 (2)
C5—C6	1.382 (3)	C13—C14	1.382 (3)
C5—H5	0.9300	C13—H13	0.9300
C6—H6	0.9300	C14—H14	0.9300
C7—O3	1.212 (2)	O1—H1	0.8200
C7—C8	1.514 (3)	O2—H2	0.8200
C8—C9	1.506 (3)	O4—H4	0.8200
C8—H8A	0.9700		
			
O1—C1—C6	124.22 (17)	C9—C8—H8B	108.4
O1—C1—C2	116.86 (17)	C7—C8—H8B	108.4
C6—C1—C2	118.92 (17)	H8A—C8—H8B	107.4
C3—C2—O2	123.43 (16)	C14—C9—C10	117.12 (19)
C3—C2—C1	120.35 (17)	C14—C9—C8	121.34 (19)
O2—C2—C1	116.22 (16)	C10—C9—C8	121.47 (19)
C2—C3—C4	121.03 (16)	C11—C10—C9	121.7 (2)
C2—C3—H3	119.5	C11—C10—H10	119.1
C4—C3—H3	119.5	C9—C10—H10	119.1
C5—C4—C3	118.43 (17)	C12—C11—C10	119.8 (2)
C5—C4—C7	123.56 (16)	C12—C11—H11	120.1
C3—C4—C7	118.00 (16)	C10—C11—H11	120.1
C6—C5—C4	120.53 (17)	C13—C12—O4	122.75 (18)
C6—C5—H5	119.7	C13—C12—C11	119.75 (19)
C4—C5—H5	119.7	O4—C12—C11	117.47 (18)
C5—C6—C1	120.75 (17)	C12—C13—C14	119.4 (2)
C5—C6—H6	119.6	C12—C13—H13	120.3
C1—C6—H6	119.6	C14—C13—H13	120.3
O3—C7—C4	120.71 (17)	C9—C14—C13	122.2 (2)
O3—C7—C8	120.36 (18)	C9—C14—H14	118.9
C4—C7—C8	118.93 (15)	C13—C14—H14	118.9
C9—C8—C7	115.66 (16)	C1—O1—H1	109.5
C9—C8—H8A	108.4	C2—O2—H2	109.5
C7—C8—H8A	108.4	C12—O4—H4	109.5
			
O1—C1—C2—C3	179.11 (17)	C3—C4—C7—C8	173.33 (18)
C6—C1—C2—C3	−0.2 (3)	O3—C7—C8—C9	11.5 (3)
O1—C1—C2—O2	−0.5 (3)	C4—C7—C8—C9	−167.73 (17)
C6—C1—C2—O2	−179.76 (17)	C7—C8—C9—C14	−121.6 (2)
O2—C2—C3—C4	−179.83 (17)	C7—C8—C9—C10	61.4 (3)
C1—C2—C3—C4	0.6 (3)	C14—C9—C10—C11	−0.6 (3)
C2—C3—C4—C5	−0.6 (3)	C8—C9—C10—C11	176.50 (18)
C2—C3—C4—C7	−179.35 (16)	C9—C10—C11—C12	0.1 (3)
C3—C4—C5—C6	0.2 (3)	C10—C11—C12—C13	0.4 (3)
C7—C4—C5—C6	178.83 (17)	C10—C11—C12—O4	−177.56 (18)
C4—C5—C6—C1	0.3 (3)	O4—C12—C13—C14	177.42 (18)
O1—C1—C6—C5	−179.49 (18)	C11—C12—C13—C14	−0.5 (3)
C2—C1—C6—C5	−0.3 (3)	C10—C9—C14—C13	0.6 (3)
C5—C4—C7—O3	175.4 (2)	C8—C9—C14—C13	−176.54 (19)
C3—C4—C7—O3	−5.9 (3)	C12—C13—C14—C9	−0.1 (3)
C5—C4—C7—C8	−5.3 (3)		

### Hydrogen-bond geometry (Å, °)

**Table:**

*D*—H···*A*	*D*—H	H···*A*	*D*···*A*	*D*—H···*A*
O1—H1···O4^i^	0.82	1.94	2.744 (2)	168
O2—H2···O3^ii^	0.82	1.91	2.7274 (19)	171
O4—H4···O2^iii^	0.82	2.00	2.772 (2)	158
C3—H3···O3^ii^	0.93	2.53	3.191 (2)	129
C11—H11···Cg1^iv^	0.93	2.85	3.635 (2)	143

Symmetry codes: (i) *x*−1, *y*−1, *z*−1; (ii) −*x*+1, −*y*+1, −*z*+1; (iii) *x*, *y*+1, *z*+1; (iv) −*x*+1, −*y*+2, −*z*+1.
